# Human papillomavirus molecular biology and disease association

**DOI:** 10.1002/rmv.1822

**Published:** 2015-03-06

**Authors:** John Doorbar, Nagayasu Egawa, Heather Griffin, Christian Kranjec, Isao Murakami

**Affiliations:** ^1^ Department of PathologyUniversity of CambridgeCambridgeUK

## Abstract

Human papillomaviruses (HPVs) have evolved over millions of years to propagate themselves in a range of different animal species including humans. Viruses that have co‐evolved slowly in this way typically cause chronic inapparent infections, with virion production in the absence of apparent disease. This is the case for many Beta and Gamma HPV types. The Alpha papillomavirus types have however evolved immunoevasion strategies that allow them to cause persistent visible papillomas. These viruses activate the cell cycle as the infected epithelial cell differentiates in order to create a replication competent environment that allows viral genome amplification and packaging into infectious particles. This is mediated by the viral E6, E7, and E5 proteins. High‐risk E6 and E7 proteins differ from their low‐risk counterparts however in being able to drive cell cycle entry in the upper epithelial layers and also to stimulate cell proliferation in the basal and parabasal layers. Deregulated expression of these cell cycle regulators underlies neoplasia and the eventual progression to cancer in individuals who cannot resolve high‐risk HPV infection. Most work to date has focused on the study of high‐risk HPV types such as HPV 16 and 18, which has led to an understanding of the molecular pathways subverted by these viruses. Such approaches will lead to the development of better strategies for disease treatment, including targeted antivirals and immunotherapeutics. Priorities are now focused toward understanding HPV neoplasias at sites other than the cervix (e.g. tonsils, other transformation zones) and toward understanding the mechanisms by which low‐risk HPV types can sometimes give rise to papillomatosis and under certain situations even cancers. Copyright © 2015 John Wiley & Sons, Ltd.

**Abbreviations used**
BCC: basal cell carcinoma
BD: Bowen's disease
cAMP: cyclic AMP
CIN: cervical intraepithelial neoplasia
CR1: conserved region 1
CR2: conserved region 2
E: early (viral genome)
HN: head and neck
HPV: human papillomavirus
L: late (viral genome)
LCR: long control region
LSIL: low‐grade squamous intraepithelial lesion
PAE: polyadenylation early
PAL: polyadenylation late
PBM: PDZ binding motif
RRP: recurrent respiratory papillomatosis
SCC: squamous cell carcinomas

## Introduction

**Figure 1:**
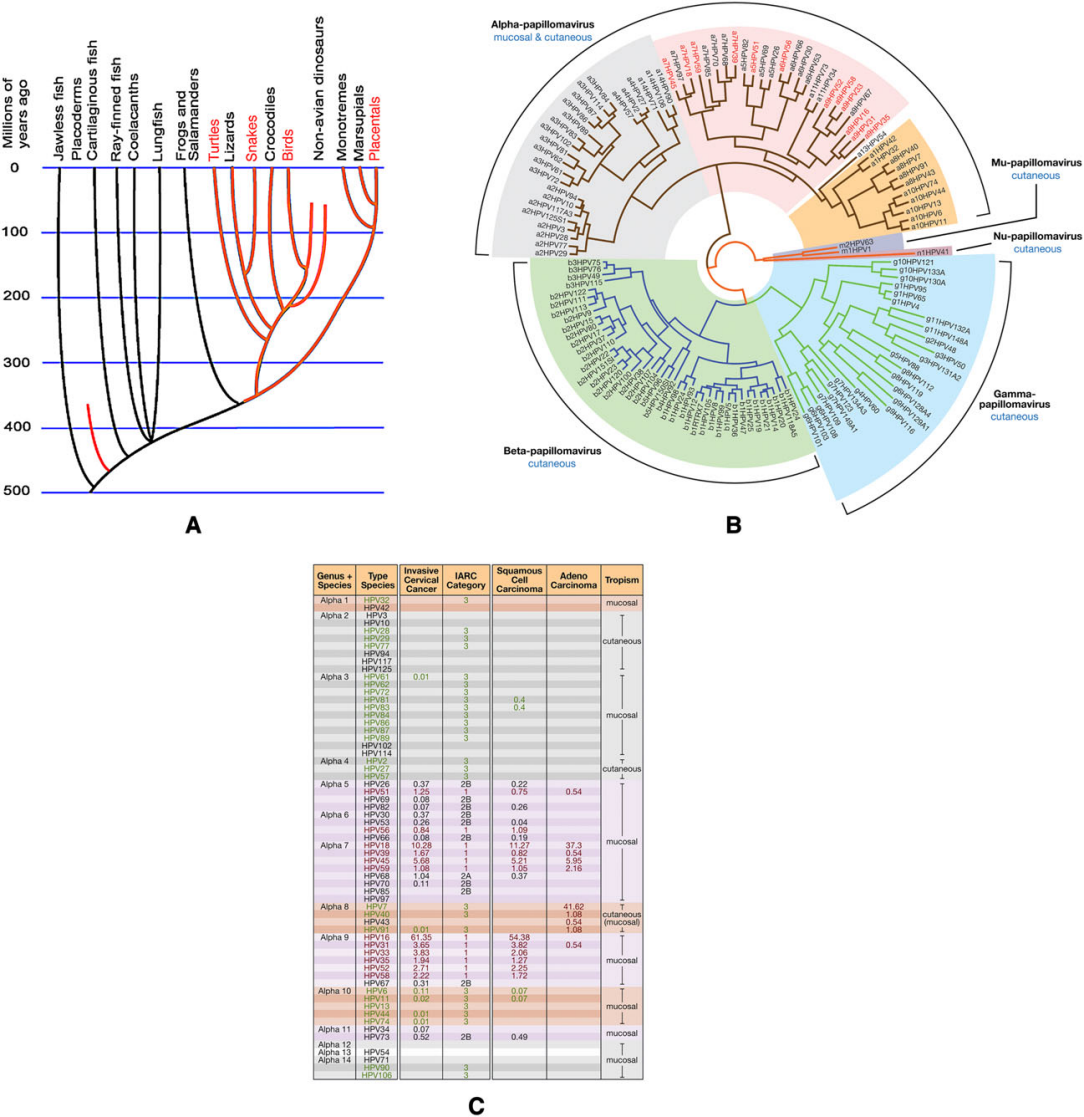
(A) Evolutionary tree showing the proposed appearance of an ancestral “papillomavirus” between the branch point leading to amphibians and reptiles. It is thought that virus/host co‐evolution has occurred during speciation, and that this has led to the widespread distribution of papillomaviruses in organisms as diverse as snakes, birds, and mammals, (B) The human papillomaviruses types found in humans fall into five genera, with the Alpha and the Beta/Gamma genera representing the largest groups. Human papillomaviruses types from the Alpha genus are often classified as low‐risk cutaneous (gray), low‐risk mucosal (orange), or high‐risk (pink). The high‐risk types identified using red text are confirmed as “human carcinogens” on the basis of epidemiological data. The remaining high‐risk types are “probable” or “possible” carcinogens. The evolutionary tree is based on alignment of the E1, E2, L1, and L2 genes 6, (C) Percentage of cervical cancers that are causally attributed to infection with members of the Alpha genus. Members of the Alpha 9 and 7 species have been studied most thoroughly

Papillomaviruses comprise a diverse group of viruses that infect both humans and animals. Their origin appears linked to changes in the epithelium of their ancestral host as the first reptiles emerged around 350 million years ago. Since then, they have co‐evolved with their respective hosts, with little cross‐transfer between species, and are now found in birds, reptiles, marsupials, and mammals, but not in amphibians or lower phylogenetic orders (Figure 1A) 1. Viruses that slowly evolve with their hosts in this way typically cause chronic inapparent infections, rather than serious disease 2. This is the case for many if not most papillomaviruses, and indeed, HPVs can be isolated from skin swabs and plucked hairs from normal immunocompetent individuals in the general population 3, 4. As a result of such observations, it is thought that many HPVs may in fact persist in the population as commensals rather than being associated with obvious disease pathology 4, 5.

The study of HPVs has been driven not by these widespread inapparent infections, but by the severity to which some HPV‐associated diseases can progress. Most significant of these is cervical cancer, which can result from persistent infection with a group of “high‐risk” HPVs 6, 7, 8. The low‐risk HPV types, although not usually associated with cancer development, can cause problematic and debilitating disease in some individuals. The association of HPV type 11 with RRP is a key example of such a disease 8. Although rare, children with RRP are unable to resolve their infection and need to be treated by repeat surgery to reduce papilloma size and to maintain a clear airway 8. At present, there is no reliable treatment for HPV infections, except by complete surgical removal of the disease site. In the case of RRP, papillomas can persist for years or decades with regular recurrence after treatment, and in some individuals, it can eventually give rise to metastatic lesions in the lower airway and lung 9.

This review aims to provide an update of current thinking regarding the mechanisms underlying lesion formation by papillomaviruses, focusing in particular on the diversity of epithelial sites that these viruses infect and the diseases that they cause. As well as outlining the basic biology of these viruses, the review aims to clarify the key differences between high‐risk Alpha papillomaviruses and low‐risk papillomavirus types from Alpha and other genera, which we hope will explain why such viruses are associated with cancers less frequently. As part of this, the different mechanisms by which Beta papillomaviruses can sometimes cause cancer are discussed.

## Papillomavirus Diversity and Epithelial Tropisms

Over 200 papillomaviruses have been identified and have been completely sequenced, including more than 150 HPV (see 10 and Papillomavirus Episteme (PaVE); http://pave.niaid.nih.gov/#home). Human types are divided into five genera based on differences in their DNA sequence, with individual types having a nucleotide sequence (sampled from the L1 gene) that is at least 10% dissimilar from that of other papillomaviruses 10. The terms “serotype” and “strain” are not used to distinguish between papillomaviruses, and indeed, many papillomaviruses have not been characterized beyond the level of their DNA sequence. In recent years, sensitive detection methods have allowed the identification of a large number of new HPV types (primarily Beta and Gamma types) from swabs taken from cutaneous epithelium or from plucked hairs http://pave.niaid.nih.gov/#home. Beta types have almost doubled in number (from 25 to 45), whereas Gamma types have increased almost eightfold (from 7 to 54) over the last decade 11. Although phylogeny provides insight into disease associations, closely related types can show distinct pathologies. HPV 6 and 11 share 85% sequence identity, but the former is found more commonly in anogenital warts than HPV 11, which is the primary cause of laryngeal papillomas. Similarly, HPV 13, which shares 78% sequence identity with HPV 6 and HPV 11, does not cause either anogenital warts or laryngeal papillomas 12, 13, whereas HPV 7, which is 87% homologous to the mucosal type HPV 40, causes “butchers” warts at cutaneous sites. Tropisms are thought to be controlled primarily at the level of viral gene expression, with regulatory elements within the long control region (LCR) being an important determinant 12. Regulation at the level of infectivity may also influence site of infection, with markedly different charge distributions being reported between cutaneous and mucosal virions 14. Successful infection requires conformational changes in the capsid, followed by furin cleavage of the minor L2 capsid protein 15, 16, 17, which may also influence the tropisms of individual HPV types 15, 16, 18, 19, 20, 21, 22. Although the diseases caused by specific HPV types sometimes occur at non‐typical sites, this is uncommon, with lesions often exhibiting non‐typical morphology and pathology 23. The evolutionary relationship between HPV types and the cancer associations of the important Alpha genus are shown in Figure 1B and C.

## Virus Structure and Genome Organization

### Virus structure

**Figure 2:**
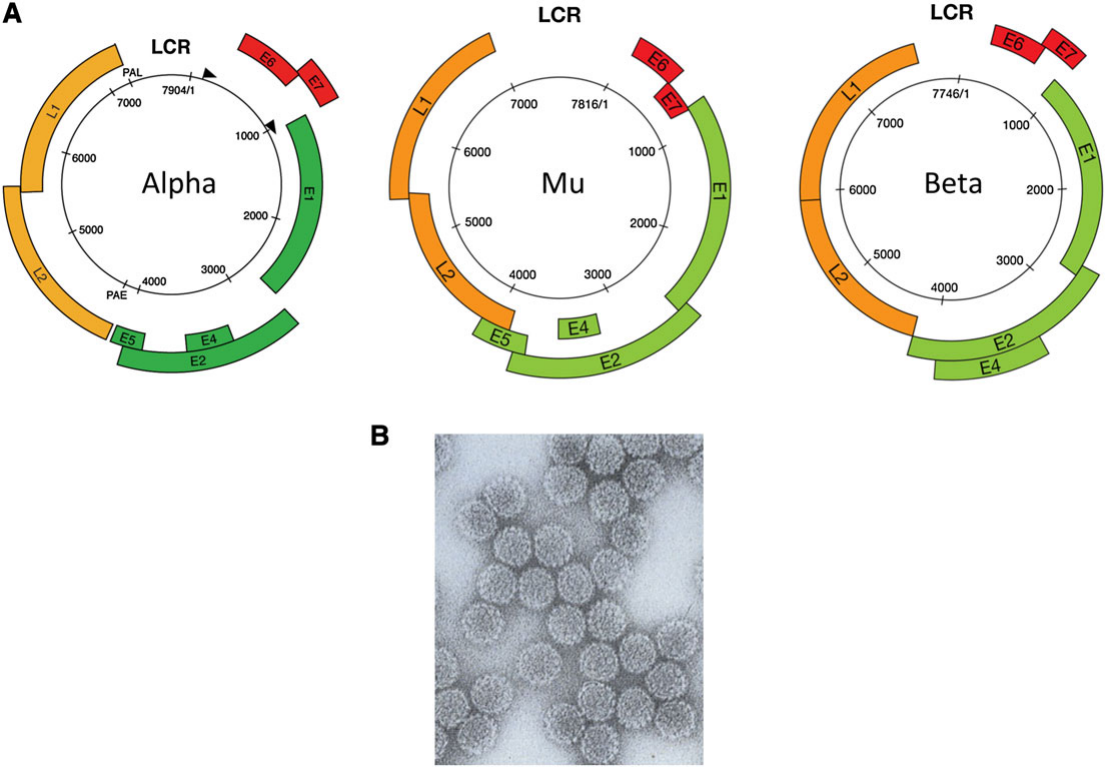
(A) Typical genome organization of the high‐risk Alpha, Mu, and Beta HPV genomes. Although all share a common genetic organization, the size and position of the major ORFs can vary, with Beta HPV types lacking an E5 ORF. The positions of the major promoters are marked with arrows on the high‐risk Alpha HPV genome map, with early and late polyadenylation sites marked as polyadenylation late and polyadenylation early, (B) Electron micrograph of negatively stained papillomavirus particles. Individual capsomeres within the capsid structure can just be visualized. Papillomavirus particles are approximately 55 nm diameter and are non‐enveloped.

Despite the different disease associations, papillomavirus particles share a common non‐enveloped icosahedral structure (50–60 nm diameter). Their genomes comprise double‐stranded circles (episomes) of approximately 8000 base pairs, which contain eight or nine ORFs. Although gene number is limited by the small size of the papillomavirus genome (Figure 2A), the number of encoded proteins is much greater, as gene expression involves the use of multiple promoters and complex patterns of splicing (http://pave.niaid.nih.gov/#home 24). The fine structure mapping 25 shows the virus coat to contain 360 molecules of L1 protein arranged into 72 capsomeres, each made up of 5 L1 molecules, which have a beta‐jellyroll core reminiscent of other icosahedral viruses (Figure 2B 26). Interactions between capsomeres require the C‐terminal tail of the L1 protein, which extends out toward neighboring capsomeres and links them at their base via disulfide bonds 26, 27, 28, 29.

Human papillomavirus particles also contain a variable number of L2 molecules, which are not fully exposed on the surface of the virion, apart from their N‐terminal 120 or so amino acids 30, 31. During infection, L2 becomes available for binding to the extracellular matrix and is cleaved by furin during the infection 16. The major surface‐exposed regions of L1 comprise a series of hypervariable amino acid loops that have diverged between different papillomavirus types, in response to host immune selection pressure, with antibodies raised to one HPV type binding to distantly related types only poorly. This has practical consequences for the current prophylactic vaccines, which offer limited cross‐protection. The virus genome also encodes regulatory proteins that stimulate cell cycle entry and cell proliferation, as well as proteins that mediate virus genome replication, virus assembly, and probably, also effective virus release and transmission. Although many of these genes are contained within the early region of the virus, the L2 gene product also has key immediate‐early functions in viral genome delivery within the cell and also a role (along with E2) in orchestrating proper genome packaging 32.

### Genome organization

Individual ORFs within the viral genome are designated early or late 11, with the lack of an E3 ORF reflecting an initial sequencing error in the BPV1 genome. Despite variation in the size and number of ORFs, all papillomaviruses contain well‐conserved core genes involved in replication (i.e. E1 and E2) and packaging (i.e. L1 and L2) with greater diversity in the remaining genes (i.e. E6, E7, E5, and E4), which have roles in driving cell cycle entry, immune evasion, and virus release 6. E1 encodes a virus‐specific DNA helicase necessary for viral genome replication and amplification, and like L1 (the major capsid protein), is highly conserved. E2, which can bind to sites in both the viral and cellular genome, is conserved between HPV types in its N‐terminal and C‐terminal domains and functions in viral transcription, replication, and genome partitioning. As with most HPV gene products, the functions of E2 are dependent on its interaction with cellular gene products and in modifying their normal roles to the benefit of the virus. The remaining genes encode proteins that modify the cellular environment or perform other functions during the life cycle of different papillomaviruses. E6 and E7 can be regulated at the transcriptional level by E2, and play a critical role in driving cell cycle entry in all HPV types to allow genome amplification in the mid‐layers of the epithelium, and to inhibit aspects of innate immunity. Interestingly, Beta papillomaviruses lack a recognizable E5 ORF, which in the Alpha genera is located downstream of E2, and which along with E6 and E7, is involved in immune evasion and in optimizing genome amplification efficiency. The E4 protein (which plays a role in virus escape from the epithelial surface), like E5, shows considerable sequence heterogeneity between types, which is thought to reflect the different tropisms and transmission routes of different papillomaviruses 33, 34. Perhaps more surprisingly, given its importance in genome amplification, the HPV E6 protein is absent in HPV 101, 103, and 108 (Gamma genera 11, 35). The papillomavirus LCR is located between the end of L1 and the start of the early region and contains promoter elements, transcription factor binding sites (including palindromic sequences recognized by E2), and the viral origin of replication (to which E1 can bind), with some animal papillomaviruses (e.g. canine oral papillomavirus) containing an additional non‐coding region between the end of the early region and the start of L2. Considerable heterogeneity exists between the positions of promoters and of splice donor and acceptor sites, which reflects the distinct evolutionary path of each HPV type 24, 34.

## Human Papillomaviruses Infection and Clinical Manifestations of Disease

### Mucosal human papillomaviruses infections

The association of HPV with cervical disease has been extensively studied. HPV detection in the absence of apparent disease is found in 11–12% of all women. Detection is higher in young women ((and men) 50–80% 36) and declines in older age groups 37. Such inapparent infections and low‐grade disease are typically characterized by multiple HPV types, including HPV 16 (3.2%), 18 (1.4%), 31 (0.8%), and 58 (0.7%). HPV detection increases with disease severity 37, with percentage positivity in CIN1/LSIL (i.e. low‐grade neoplasia) of between 50–70%. In CIN2, there is 85% positivity for HPV and in CIN3 and invasive cervical cancer; the positivity rises to between 90% and 100% 38. The detection of high‐risk HPV types at other sites varies and in the oral cavity is estimated at around 5% in apparently asymptomatically infected individuals 39, rising to 50% or so in individuals with oropharyngeal cancers 40. Although genital warts are typically benign lesions, the incidence of new cases per year in UK is 0.16%, with an incidence of recurrent cases of 0.13% 41. These figures underlie the prevalence of low‐risk genital HPV infections and the difficulties in reliably eliminating them with current treatment. HPVs produced from genital warts are associated with a transmission rate of 60%, and like high‐risk infections, are most prominent in the late teens and early 20s 42. Pathology and HPV‐type associations of important mucosal lesions are described in box 1.

**Box 1**

KEY FACTS—Mucosal Papillomavirus Infections in Humans

*Condyloma acuminatum* is one of the most common manifestations of HPV in the genital area 43. They present as papules, nodules or soft, filiform, pinkish, sessile or pedunculated growths. In men, genital condylomas more commonly involve the coronal sulcus, the glands penis, and the penile shaft. In women, lesions commonly affect the external genitalia and the cervix 44. The disease is usually sexually transmitted and is most frequently caused by low‐risk HPVs, such as HPV 6 and 11, although many other genotypes can also be found, including HPV 2, 16, 18, 30–33, 35, 39, 41–45, 51–56, and 59 45, 46, 47. As described in the text, the HPV types that cause benign genital warts can also cause problematic papillomas at oral sites, which can be difficult to treat because of their location.
*Focal epithelial hyperplasia* is a rare HPV‐related disease of the oral mucosa that is more common in children and women. Lesions are mainly located in the lower lip, but less frequently may affect the upper lip, tongue, oral mucosa, oropharynx, palate, and floor of mouth. HPV 13 and 32 are the most common cause 48.
*Cervical neoplasia and cervical cancer*. Precancerous cervical lesions are classified as cervical CIN of different grades (1, 2, or 3). CIN1 pathology is broadly equivalent to the LSIL designation used in the Bethesda classification system, with CIN2 and 3 being equivalent to high‐grade squamous intraepithelial lesion. The severity of neoplasia reflects the extent to which basal‐like cells (i.e. poorly differentiated cells with a high nuclear/cytoplasmic ratio) extend toward the epithelial surface and the extent of suprabasal cell division. Low‐grade lesions typically show evidence of productive viral infection with the presence of koilocytes in the suprabasal cell layers being regarded as a key manifestation of CIN1/LSIL. HPV is detectable in 90–100% of cervical abnormalities, ranging from incipient cytological abnormalities and dysplasia 49 to cervical cancer 50, 51, 52.
*Other anogenital cancers* including those of the vulva, vagina, penis, and anus. Most vulvar cancers (92%) are solitary, keratinizing SCC. HPV prevalence is 90% in vulvar intraepithelial neoplasia and basaloid or warty cancers, but is found in only 6% of keratinizing SCC 53, 54. HPV 16 is the most prominent type in vulvar cancer, with HPV 18, 21, 31, 33, and 34 detected at lower frequencies. In addition, HPV is responsible for 85% of vaginal cancer, with HPV 16 being detected in 60% of invasive tumors. HPV is also detected in basaloid and warty cancers of the penis, but only rarely in keratinizing SCC and verrucous cancers of the penis. In invasive penile cancer, HPV 16 is the most prevalent type (40–70%), followed by HPV 6 (22%), 52 (15%), and 11 (4%) 55. HPV is present in 80–96% of anal cancer with HPV 16 being the most prevalent type 56. Anal cancer is more common in men who have sex with men, individuals with a history of anal warts, and in immunosuppressed populations.
*Head and neck cancer* HPV is recognized as a major risk factor for the development of HNSCC. A recent meta‐analysis showed that HPV prevalence in HNSCC increased significantly from 41% in 2000 to 72% in 2004 57. HPV prevalence is significantly higher in oropharynx SCC than in the oral cavity with the tonsil having higher prevalence than other anatomic sites 58. These HPV‐associated cancers display clinical and molecular features distinct from other HNSCCs. The patients with HPV‐positive cancer have at least a 50% improvement in overall survival at 5 years, which is equivalent to an approximate 30% difference in absolute survival. HPV association is now part of routine diagnostic procedure when assessing the prognosis of HNSCC. HPV 16 is the most common type found in HNSCC, but other HPV types such as 18, 31, 33, and 35 can also be detected 57.

### Cutaneous human papillomaviruses infections

Among the HPV types associated with cutaneous disease are HPV types 2, 3, 10, 27, and 57 from the Alpha Genus, HPV types 4, 60, and 65 from the Gamma Genus, and HPV types 1 and 63 from the Mu Genus. Such benign lesions are relatively common in the general population, particularly in children (33% positive) who may be encountering HPV types for the first time and in immunosuppressed individuals (45% positive) 59. An incubation period of 3 weeks to 8 months can occur before lesions become apparent, depending on inoculation titre 60. The Alpha types (2, 27, and 57) are most prevalent in common warts (>65% of cases), along with HPV 1 (Mu HPV type; approx. 30% of cases) 59. In most cases, such lesions are an inconvenience with spontaneous immune regression of 80% within 2 years 61. Benign warts such as these can be highly productive and contain as many as 10^12^ particles 62 and typically show general hypertrophy (cell enlargement) leading to acanthosis or thickening of the epithelium, as well as prominent folding of the epithelial basal layer (papillomatosis). Such lesions have thicker cornified layers (hyperkeratosis) and contain abundant cytoplasmic inclusion granules of characteristic appearance in the spinous and granular layers, which comprises predominantly of the viral E4 protein 34. Virions released from the epithelial surface may be transmitted indirectly (e.g. on innate objects) or directly from person to person 63. The pathology features and type associations of the most prevalent benign cutaneous lesions are described in box 2.

**Box 2**

KEY FACTS—Cutaneous Papillomavirus Infections in Humans

*Common warts* can be single or multiple and of varying sizes. They occur at many sites, but often on the back of hands 64, with the knee also being a common site of infection in children. A prevalence of 3.5% 65 in adults to over 30% in schoolchildren has been reported 66. Incidence increases in immunosuppressed patients, with lesions being more numerous and more recalcitrant. HPV 1, 2, 4, 27, and 57 are most prevalent types 67, 68, 69]. HPV 7 is found in the common warts of individuals whose hands are chronically exposed to moisture and cold because of their occupation 70.
*Plantar warts* occur on the soles of the feet, particularly in children. HPV 1 and 4 are frequently the cause, although HPV 57, 60, 63, 65, and 66 can also be involved 68. HPV 1 commonly induces lesions that manifest as a keratotic plug surrounded by a hyperkeratotic rim that are often painful. HPV 4 can be the cause of mosaic warts, which are more superficial lesions that occur in a confluent cobblestone pattern and are usually painless. Persistent plantar lesions can be very rarely associated with the development of verrucous carcinoma 72.
*Flat warts* are slightly raised lesions of skin color or pigmented, with flat, smooth or, slightly rough surface. The face and back of hands are the most common sites of disease with HPV 3 and 10 most commonly detected in such lesions 64, 73.
*Filiform warts* are pedunculated lesions growing in a perpendicular or oblique way in relation to the skin surface. The face and neck are the most frequent sites of disease. The detected HPV types are the same as common warts, especially HPV 2 73.
*Pigmented warts* range from gray to blackish brown and are located on the palmoplantar or lateral surfaces of the hands, feet, fingers, and toes. HPV 4, 60, and 65 are most prevalent in such lesions 74.
*Epidermoid cysts* can be caused by HPV types 57 and 60, with these types being detected in plantar epidermoid cysts 75, 76. An unknown HPV type was reported in epidermoid cysts of the trunk and scalp 77, 78. Immunostaining suggests that such lesions are distinct from the associated dermal eccrine duct, but have similarities with the suprabasal cells of the epidermis. It has been suggested that palmoplantar epidermoid cysts may in some instances arise as a result of epidermoid metaplasia of eccrine ducts following HPV infection 79.
*Skin cancer.* Bowen's disease (BD) is a SCC *in situ* of the skin. In 3–5% of cases, it progresses to invasive carcinoma with the capability to develop metastasis. The mucosal HPV types are commonly detected in lesions of extra‐genital BD, especially in the periungual region. Other HPV types have occasionally been detected in BD, including HPV 2, 6, 11, 54, 58, 61, 62, and 73 80. The link between HPV and non‐melanoma skin cancer, SCC and BCC, is not clear except in immunosuppressed individuals and in certain genetic backgrounds. Mucosal HPV types, especially HPV 16 can sometimes be detected in the SCC and BCC of the skin, but also more rarely HPV 2, 31, 34, 35, 58, 61, and 73 81, 82. Molecular analysis of Beta HPV protein function and serology suggests a role of certain Beta HPV types (e.g. HPV 8, 20, 38) in the development of SCC in immunosuppressed individuals. A role in the early stages of cancer development is suspected (but not conclusively proven) in a fraction of keratinocyte cancers in the general population, with Beta HPV genomes from the cell being lost from the cell as the disease severity increases 83.

## Papillomavirus Life Cycle Organization in the Infected Epithelium

The ability of specific HPVs to undergo a productive life cycle depends on the site of infection as well as the local microenvironment 84. Although HPV life cycle organization is best understood for Alpha papillomavirus, the broad principles are likely to be common to HPVs in general. In many cases, lesion formation is thought to begin with a wound or other epithelial trauma followed by the infection of an epithelial basal stem cell, with the longevity of these cells underlying lesion persistence 85, 86, 87, 88. For low‐risk HPV types, which do not stimulate cell proliferation, this is a reasonable hypothesis 89, 90, 91, 92, 93. For the high‐risk types, which can drive cell proliferation, it is less clear. Active cell division (as it occurs during wound healing) is necessary for viral genome entry into the nucleus and episomal maintenance 94. The particular susceptibility of the cervical transformation zone to cancer progression may be linked to increased likelihood of infection, particularly at puberty when metaplastic cells are present at this site 95, 96, 97. Recent studies have suggested the presence of cuboidal stem‐like cells at the squamo‐columnar junction, which may be prone to cancer progression following infection by high‐risk HPV types 98.

### Infection and genome maintenance in the epithelial basal layer

**Figure 3:**
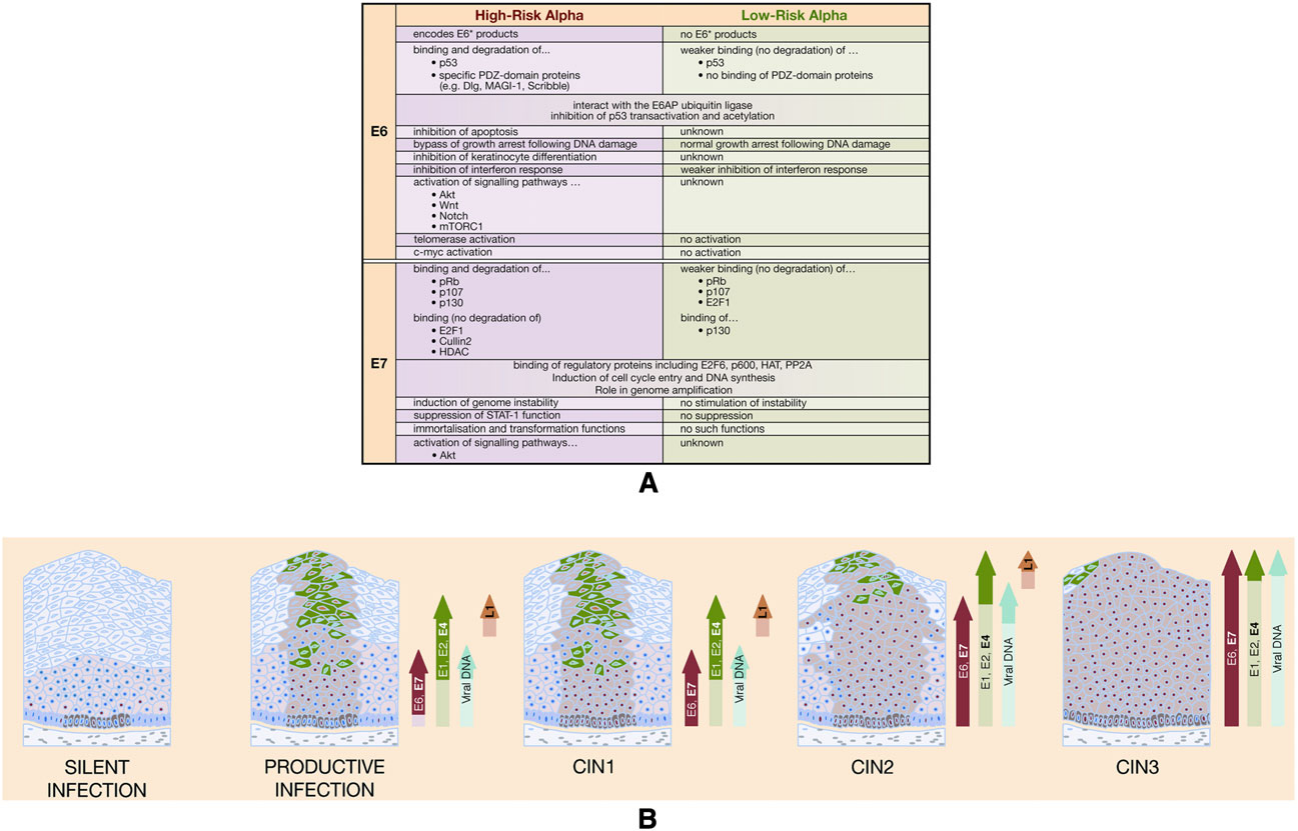
(A) The E6 and E7 proteins of the high‐risk and low‐risk HPV types have different functions, which reflect their different biologies. The ability of the high‐risk HPV types to drive cell division in neoplasia is thought to reflect the ability of their E7 protein to bind and degrade multiple members of the pRb protein family, as well as the ability of E6 to efficiently degrade p53 and to compromise the function of PDZ‐domain proteins that regulate cell contact and signaling pathways, (B) High‐risk HPV infection can lead to a “silent” or asymptomatic infection in which viral genomes persist in the basal layer without the development of disease, or alternatively to the development of a productive lesion such as CIN1 in which viral gene expression is regulated as the infected cells differentiate. In some instances, infection can lead to higher‐grade neoplasia, with deregulated viral gene expression leading to secondary genetic changes in the host cell and possible integration of the viral genome into the cellular chromosome. The deregulated gene expression seen in CIN2 and 3, which are considered to be precancerous lesions, predispose to the development of cancer

Infection is thought to be followed by an initial phase of genome amplification, prior to maintenance of the viral episome at low copy number 94, 99. Episomal copy number in the infected basal cell is often quoted as 200 copies per cell, based on a study of cell lines. Using laser capture methods, 50–100 copies per cell 100 have been found in the basal layer of productive warts. The viral replication proteins E1 and E2 are important for this initial amplification phase, but may be dispensable for episomal maintenance–replication once the copy number has stabilized 101, 102, 103 despite established roles for E2 in genome partitioning, replication, and transcription 85, 104, 105. In BPV, genome partitioning upon cell division involves the cellular bromodomain containing protein 4(Brd4), but in HPVs, other E2 binding proteins may also be involved in the tethering of viral episomes to the cellular chromatin during cell division 106, 107, 108, 109. Interestingly, Brd4 has been implicated as a key protein involved in HPV 16 genome replication 110. The E6 and E7 proteins are key regulators of cell cycle progression, but their precise role in infected basal cells is somewhat uncertain, particularly for the low‐risk HPV types (such as HPV 6 or 11) that are not generally associated with neoplasia, and which may require infection of a basal stem cell at the site of a wound or microwound. In these HPV types, the role of the wound healing response in driving the initial proliferation of the infected cell(s) is thought to be critical 111, with signaling from the local microenvironment influencing viral gene expression 112 and/or protein functions. In the case of the high‐risk types that cause neoplasia, a clear role exists for the viral E6 and E7 proteins in driving cell proliferation in the basal and parabasal cell layers, especially at sites (such as the cervix) where neoplasia can occur 85. Functional differences in E6 and E7 that are thought to underlie high‐risk and low‐risk disease pathology are listed in Figure 3A 50, 113, 114.

### Cell cycle entry and genome amplification in the suprabasal layers

The E6/E7‐mediated proliferation of basal/parabasal cells following infection by the high‐risk HPV types allows an expansion in lesion size. An important difference between high‐risk and low‐risk E7 proteins is their differential ability to associate with the retinoblastoma protein (pRb) and more specifically, the ability of the high‐risk E7 to bind and degrade p105 and p107, which control cell cycle entry in the basal layer, as well as p130, which is involved in cell cycle re‐entry in the upper epithelial layers 115, 116, 117 113, 117 (Figure 3B). These key differences between high‐risk and low‐risk E7 proteins reside in their N‐terminal half, a region that shares homology with CR1 and CR2 of the adenovirus E1A and Simian vacuolating virus 40 T‐antigen proteins 118. The biological activities of adenovirus E1a, including pRb binding and the ability to cooperate with *ras* to transform primary rat cells map to this region 119, with the pRb‐binding motif (LXCXE) being located in the CR2 region of both high‐risk and low‐risk mucosal E7 proteins. The expression of the high‐risk E7 protein leads also to an extensive epigenetic reprogramming of the cell, which is also considered important for stimulation of cell‐cycle entry and progression by E7. HPV 16 E7 interacts with Mi2*β*, a component of the nucleosome remodeling and deacetylase complex (NuRD complex), an association that is thought to block the activity of histone deacetylases 1 and 2 120. Interaction requires the C‐terminal zinc‐finger domain of E7 and contributes to the transcription of E2F‐responsive genes and the repression of pRb‐induced quiescence 120, 121. In addition, E7 expression stimulates the activation of EZH2, a histone methyl transferase, and also the histone demethylases KDM6A and KDM6B 122, 123 through different mechanisms. Interestingly, the activation of KDMs appears to be involved in the induction of p16^INK4A^, a surrogate biomarker of HPV infection, as well as homeotic genes of the *HOX* family, which have been shown to negatively regulate epidermal differentiation 124, 125. Interestingly, the effects on EZH2 are conserved between high‐risk and low‐risk E7 proteins and provide a link between viral gene expression and the modulation of events during the viral life cycle.

The PDZ (*P*SD95/*D*lg/*Z*O‐1) binding motif (PBM), which is located at the extreme C‐terminus of the high‐risk E6 proteins, represents another key difference between high‐risk and low‐risk papillomavirus types 126, 127. The E6 PBM facilitates interaction with a panel of PDZ domain‐containing proteins, and in many cases leads to their proteasome‐mediated degradation in an E6AP‐dependent manner 128, 129. So far, 14 E6 PDZ domain‐containing substrates have been identified 130, 131, with many of these (i.e. Dlg1, Scribble, MAGI‐1, ‐2, ‐3) being involved in the assembly of signaling complexes associated with the regulation of cell polarity, cell adhesion, and differentiation (reviewed in 132). Although the importance of E6‐PDZ associations has primarily been studied in the context of HPV‐related carcinogenesis, the PDZ‐binding activity of E6 appears also to regulate multiple aspects of the viral life cycle. The integrity of the E6 PBM is required for the episomal maintenance of HPV 31 and HPV 16 genomes in primary human keratinocytes 133, 134, 135 and disruption of the E6 PBM correlated with defective HPV 18 genome amplification and S‐phase re‐entry in differentiating epithelium 136. Such studies have also suggested a role for E6 PDZ‐binding activity in the expression of cyclin B1, which is required for normal G2‐M transition 137, 138. Interestingly, several high‐risk E6 proteins contain canonical cyclic AMP‐dependent protein kinase A recognition motifs (R‐R/K‐X‐T/S) within their PBM that can regulate E6 function during the high‐risk HPV life cycle 136, 139. Mechanistically, it is thought that phosphorylation regulates E6 binding to PDZ‐domain proteins and creates an alternative binding‐site, which allows E6 to associate with members of the cellular 14‐3‐3 protein family 139, 140. The high‐risk E6 proteins are also characterized by an ability to upregulate telomerase activity 141, 142, 143 and to maintain telomere integrity during repeated cell divisions, as well as by their ability to mediate p53 degradation within the cell. Both high‐risk and low‐risk E6 proteins inactivate p53 function, which suggests an important role in the virus life cycle, but only high‐risk types stimulate its ubiquitination and proteosome‐dependent degradation (see section on cancer progression below) 144, 145, 146. The high‐risk types use degradatory pathways to target several of their substrates. For E7, this is mediated via the cullin 2 ubiquitin ligase complex, whereas for E6, it involves the E6AP cellular ubiquitin ligase 147. It is now clear that both E6 and E7 have a very large number of cellular substrates, and that the identity of these substrates differs between HPV types of the same high‐risk species, as well as between the broader high‐risk and low‐risk groupings 148. The difficulty in linking defined protein functions to HPV cancer risk and indeed life cycle events is exemplified by the shared ability of high‐risk E6 proteins to degrade p53 and PDZ substrates and to induce keratinocyte immortalisation. For E6, recent structural studies have suggested a complex multimeric protein that has potential to associate with multiple protein partners at any given time point 145, 149.

In the virus life cycle, the E6 and E7 play an essential role in driving S‐phase re‐entry in the upper epithelial layers to allow viral genome amplification. This also requires the E1 and E2 proteins, which increase in abundance following “late” promoter upregulation (p670 in HPV 16; 150) in cells, which continue to express E6 and E7 from the early promoter (p97 in HPV 16). In the case of low‐risk HPV types, genome amplification requires cell cycle re‐entry in the mid to upper epithelial layers rather than occurring in cells that have remained in cycle after leaving the basal layer. For both high‐risk and low‐risk HPVs, genome amplification persists as the infected cell moves from an S‐phase to a G2‐ like phase before committing to full differentiation 151, 152.

Experimental systems show a two‐log increase in viral copy number per cell during genome amplification 100. In addition to E1 and E2, the E4 and E5 proteins also contribute to genome amplification indirectly. E5 is involved in koilocyte formation 153 and is a three‐pass transmembrane protein with a cytoplasmic C‐terminus 154. The E5 protein has a pore‐forming capability and can interfere with apoptosis 155 and the intracellular trafficking of endocytotic vesicles 156, 157. It is thought that E5 contributes to genome amplification through its ability to stabilize epidermal growth factor receptor, to enhance epidermal growth factor signaling and mitogen‐activated protein (MAP) kinase activity 158, 159, 160, 161, and also to modulate both extracellular‐signal‐regulated kinase 1/2 (ERK 1/2) and p38 independently of epidermal growth factor receptor 162, 163. The cellular MAP kinases ERK 1/2 regulate nuclear E1 accumulation through the phosphorylation and activation of a nuclear localisation signal within the E1 protein, with their activity being dependent on upstream MAP kinase kinase 1/2 (MEK, MAPKK) and p38. The accumulation of cyclin E and A and their associated cyclin‐dependent kinase 2 in S‐phase further contributes by phosphorylation and inhibition of the nuclear export sequence of E1 164, 165. Other post‐translational modifications in E1 (e.g. cleavage by caspases) may also facilitate differentiation‐dependent genome amplification, with the accumulation of E1 in the nucleus enhancing viral DNA replication at the expense of cellular replication through induction of a DNA damage response 166. The E4 protein, which accumulates at very high levels in cells supporting virus synthesis 167, 168 may in fact have a primary function in virus release or transmission 169, 170, with the optimization of genome amplification occurring as an indirect consequence of its expression 34, 171, 172, 173, 174, 175.

### Virus assembly and virus release from the epithelial surface

Completion of the HPV life cycle ultimately involves expression of the minor coat protein (L2), cell cycle exit, and the expression of the major coat protein L1 to allow genome packaging. This requires a change in splice site usage rather than the activation of new promoters, leading to an elevation in transcripts that initiate at the late promoter (p670 in HPV 16) and which terminate at the late (rather than the early) polyadenylation site 85, an event that is facilitated by the higher levels of E2 expression that down‐regulate p97 176, 177. These changes result in a switch from the production of an E1^E4, E5 mRNA to an E1^E4, L1 transcript as genome amplification gives way to genome packaging 177, 178, 179. Encapsidation of the viral genome ultimately involves the recruitment of L2 (by E2) to regions of replication prior to the expression of L1 and the assembly of the infectious virions in the nucleus 180, 181. Virus maturation eventually takes place in the superficial dying keratinocytes, which lose mitochondrial oxidative phosphorylation and convert from a reducing to an oxidizing environment before virus release. This enables the progressive accumulation of disulfide bonds between the L1 proteins, leading to the production of stable infectious virions 182, 183. The abundant E4 protein assembles into amyloid fibrils that disrupt keratin structure and compromise the normal assembly of the cornified envelope 168, 170, 184. Although not precisely defined, it is thought that E4 amyloid fibers may contribute to virion release and infectivity in the upper epithelial layers.

## High‐Risk and Low‐Risk Human Papillomavirus Types and the Development of Cancer

**Figure 4:**
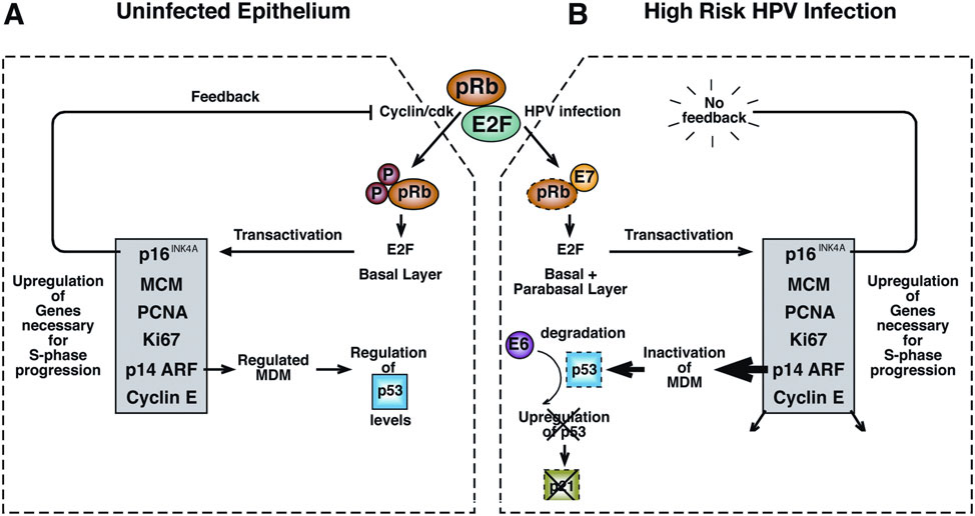
High‐risk human papillomaviruses (HPV) infection disrupts the molecular pathways that regulate epithelial differentiation and cell proliferation. Cell cycle progression is regulated in the different epithelial layers by members of the pRb (retinoblastoma) family of proteins. The E7 proteins of high‐risk HPV types can target members of this protein family for degradation (shown in **B**). This releases members of the E2F transcription factor family, which allows basal and parabasal cells to enter S‐phase. In uninfected epithelium (shown in **A**), the release of E2F is dependent on external growth factors, which stimulate cyclinD/cdk activity to allow pRb phosphorylation and E2F release. The expression of cellular proteins involved in cell cycle progression is regulated by p16^INK4A^, which is involved in a negative feedback loop by suppressing the activity of the cyclinD/cdk. The inability of low‐risk HPV types to drive robust basal cell proliferation is thought to be because these types can only efficiently target the p130 retinoblastoma family member, which controls suprabasal, but not basal cell cycle entry. The high‐risk E7 proteins are thought to target all members of the pRb family. In addition to E7, high‐risk HPVs encode a second protein involved in cell cycle entry. This is the E6 protein, which acts to suppress the rise in p53 that would otherwise occur following E7‐mediated elevation in p14 levels. (shown in **B**) Elevated p14 leads to inactivation of the MDM protein that is normally involved in degrading p53. High‐risk E6 proteins directly regulate p53 levels in the cell by mediating its ubiquitination and degradation via the proteasome pathway. In uninfected cells (shown in **A**), p53 levels are maintained at a low level, partly as a result of the normal activity of MDM

The ordered expression of viral gene products that leads to virus particle production is disturbed in HPV‐associated neoplasias (Figure 3B). In cervical disease, it is thought that the levels of E6 and E7 expression rise from CIN1 to CIN3, and that these changes in gene expression underlie the different neoplastic phenotypes, with CIN1 lesions typically supporting the complete HPV life cycle 185. The increase in E6 and E7 activity that is thought to occur in high‐risk HPV infection underlies the CIN2+ phenotype and predisposes the cell to the accumulation of genetic errors that eventually lead to cancer progression 114. In this model, the lower E6/E7 activity in CIN1 is not expected to compromise the functions of the cellular targets of E6 and E7 sufficiently to facilitate cancer progression. The deregulated expression of high‐risk E7 proteins can stimulate host genome instability through deregulation of the centrosome cycle 186, 187, 188, 189, 190, 191, whereas deregulated expression of E6 contributes to the accumulation of mutations by compromising the role of p53 in DNA repair. p53 is important for the induction of cell cycle arrest and apoptosis upon aberrant cell cycle progression and is a target of both high‐risk and low‐risk E6 proteins, which act to counter the rise in p53 that results from the unscheduled DNA synthesis mediated by E7 (Figure 4). Indeed, recent studies using HPV 18 organotypic raft culture systems show that the loss of E6 and the accumulation of p53 lead to a severe impairment of the productive stage of the viral life cycle 152, 192. A similar dependency on p53 inactivation is also expected in the low‐risk HPV life cycle given the ability of these viruses to promote cell cycle re‐entry in the parabasal layers of the epithelium. Interestingly, high‐risk and low‐risk mutant HPV genomes encoding E6 proteins defective in p53 binding cannot maintain episomal genomes 192, 193, suggesting that the inactivation of p53 plays important and pleiotropic roles within the HPV life cycle. Both high‐risk and low‐risk mucosal HPV types inhibit the p300/CBP (CREB‐binding protein) mediated acetylation of p53 that is required for promoter activation 194, 195 via a mechanism that involves the formation of a complex between the histone acetyltransferase, p53, and E6, but which does not depend on the E6 associated protein 194. Low‐risk HPVs may also interfere with p53 function by mediating its cytoplasmic sequestration 196. Unlike low‐risk HPV types, however, the high‐risk E6 protein can promote the E6 associated protein‐mediated degradation of p53 and also of hAda3 (human homologue of yeast alteration/deficiency in activation 3), which is a p53 coactivator and a component of the histone acetyltransferase complex 197, 198. These functions of E6 and the other characterized functions of E6 and E7 described in the context of the virus life cycle earlier, underlie the ability of the high‐risk HPV types to cause cancers. Deregulation of viral gene expression in CIN2/3+ facilitate integration of the viral episome into the host cell chromosome, which can act to further deregulate the expression of E6 and E7. Although it is unclear how gene expression from the viral episome becomes deregulated in early CIN, data from the vaccine trials indicate that CIN2+ can sometimes occur in young women soon after infection 199, 200, 201, 202. The deregulated viral gene expression that is thought to underlie the CIN2 phenotype may be driven by hormonal changes, which affect the proliferative capacity of the infected cell 84 and/or by epigenetic modifications, which may depend on the nature of the infected epithelial cell 203. Interestingly, the HPV 16 LCR contains hormone response elements that can be stimulated by estrogen, and there is considerable evidence of cooperation between estrogen and HPV in the development of cervical cancer in humans and in model systems 84, 204, 205, 206. Several studies have recently reported that the LCR is differentially methylated according to disease grade, which suggests that epigenetic changes may also regulate promoter usage 207 (and thus disease 114) and indeed be exacerbated by the expression of the viral oncogenes 122, 208, 209. Although common fragile sites in the host genome are considered to be hot spots where integration is likely to occur 210, integration is a chance event, which can sometimes result in disruption of the viral E2 gene that normally suppresses transcription of E6 and E7. Most cervical cancers contain one or more copies of HPV integrated into the host chromosome, with the viral integration site frequently lying either within the E1 or E2 open reading frame 211, 212. Integration and the loss of normal E6/E7 regulation by E2 facilitates long‐term/ high‐level expression of these genes 213, 214, 215, and generally occurs in high‐grade lesions such as CIN2 and CIN3 86 216 (Figure 3B). Cervical cancer can arise from cells containing exclusive episomes, and for HPV 16, around 30% (between 26 and 76% depending on study) of cervical cancers develop in this way 217, 218, 219. Approximately 70% of HPV 16‐associated cervical cancers contain integrated HPV 16 sequences, whereas for HPV 18, the viral genome is nearly always integrated 220, 221, 222, 223, 224.

## Host Immune Responses in Lesion Regression and Clearance

Although high‐risk HPV infection is common, with over 80% of women becoming infected at some stage in their life, cervical cancer arises rarely as a result of infection. Most infections are cleared by a cell‐mediated immune response, although HPV 16 and 18 persist longer than other high‐risk types, which may contribute to their higher cancer risk at stratified and glandular sites 225, 226, 227. In general however, genital tract infections by HPV are common in young sexually active individuals, with the majority (80–90%) clearing the infection without clinical symptoms. Regression of anogenital warts is accompanied by a CD4+ T cell‐dominated Th1 response, which is also seen in animal models of papillomavirus disease 228, 229, 230, 231, with a failure to develop an effective cell‐mediated immune response correlating with persistent infection, and for high‐risk HPVs, an increased probability of progression toward invasive carcinoma.

**Figure 5:**
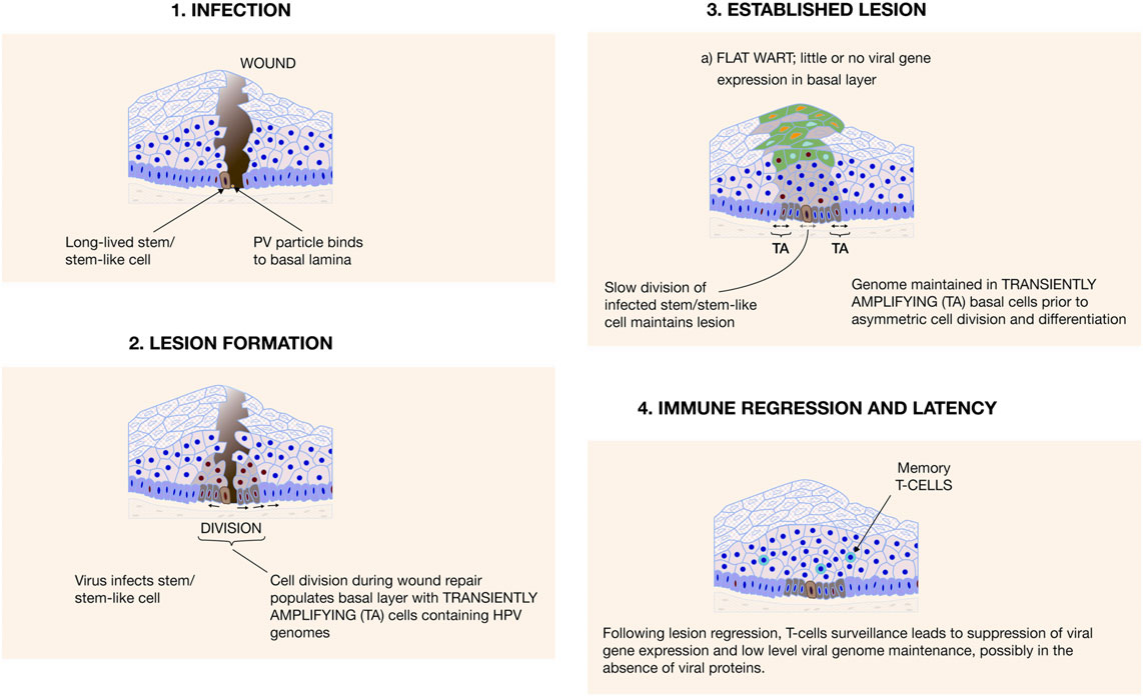
Lesion formation is thought to be facilitated by the presence of microwounds, which allows the virus to infect epithelial basal cells (e.g. an epithelial stem cell (1)). At particular sites, such as the squamocolumnar junction of the cervical transformation zone, basal cells, reserve cells, and stem‐like/stem cells are close to the epithelial surface and may be more prone to infection. At other sites, the development of a lesion may be facilitated by a wound repair (2). Once a lesion has become established, basal and parabasal epithelial cells can be driven into the cell cycle, either to mediate basal cell division (i.e. cell proliferation) or to drive cell cycle re‐entry (but not mitosis) in the upper epithelial layers in order to support viral genome amplification (3). Clearance of disease involves activation of a cell‐mediated immune response and a suppression of viral gene expression as activated T‐cells accumulate in the vicinity of the lesion. It is thought that viral genomes can persist in the basal epithelial cells with very limited gene expression, allowing possible reactivation under some circumstances, such as it can occur following immunosuppression 245

In addition, many HPV infections counter detection by the innate immune response. The life cycle is intra‐epithelial, produces no viraemia, cell lysis, or cell death, and replication is not associated with inflammation 232. Pro‐inflammatory cytokines such as Type I interferons are not released, and the signals for Langerhans cell/dendritic cell activation, migration, and recruitment are largely absent 233. Productively infected cells expressing abundant viral proteins are shed from the surface of the epithelium, away from circulating immune cells. For high‐risk Alpha types, several mechanisms of immune evasion have been established. The E6 protein of high‐risk HPV types is known to interfere with Tyk2 function, and as a result of this is thought to affect STAT signaling 85, 234, 235. Similarly, E7 is able to interfere with the induction of interferon response factor 1, with both E6 and E7 being reported to reduce surface levels of E‐cadherin, which is thought to underlie the lower abundance of Langerhans cells (the epithelial dendritic cells) in lesional tissue 236, 237, 238, 239. E7 reduces the total MHC abundance at the cell surface, and through its effects on STAT1 signaling and the suppression of IRF‐1, also reduces the levels of MHC 1 antigen presentation, which is expected to contribute to immune escape in high‐risk HPV driven cancers 240, 241, 242. Interestingly, the high‐risk E5 protein also interferes with classical MHC class 1 processing and is thought to compromise the display of viral peptides at the surface of the infected epithelial cell during the normal productive life cycle 243. The low level presentation of viral antigens, in conjunction with active immune evasion strategies and the absence of inflammation, is thought to favor immune tolerance rather than an effector T cell response able to clear disease. Resolution of infection is thought to require cross‐priming of dendritic cells with viral antigens, followed by T‐cell infiltration into the site of infection and shut‐off of viral gene expression. Human Langerhans cells are known to prime and cross prime naive CD8+ cells 244 although recent data from the mouse 49 suggest that in the skin, the important cross presenting antigen presenting cells are Langerin‐positive and CD103‐positive dendritic cells, which may be of dermal origin. When lesion regression does occur, it is not associated with significant apoptosis or cell death, and it appears from animal model studies that lesions are cleared by the replacement of actively infected cells with “apparently normal cells” as the basal cells continue to divide 100, 230, 245. These “normal” cells can still contain viral genomes but without obvious viral gene expression, with the virus life cycle becoming “re‐activated” subsequently following immune suppression or possibly also upon changes in hormone levels (Figure 5, [245). For cancer to develop, the virus has to evade immune detection over a prolonged period of time. Cervical cancer patients have a reduced or non‐existent T‐cell response to antigens of the causal HPV type 246, 247, which suggests that persistence may be linked to a failure of the immune response or an inability to recognize viral antigens. No obvious link between HLA type or other susceptibility indicators has however yet been made 248, 249, 250.

## Conclusions

Human papillomaviruses have evolved over many millions of years to propagate themselves in a range of different animal species including humans. A typical characteristic of viruses that have co‐evolved with their hosts in this way is the production of chronic inapparent infections, with virion production from the surface of infected epithelium in the absence apparent disease. This is the case for many Beta and Gamma HPV types. However, not all HPV types use this approach, and it appears that several Alpha papillomavirus types have evolved immunoevasion strategies that allow them to cause persistent visible papillomas. As part of the papillomavirus life cycle in the epithelium, these viruses need to activate the cell cycle as the infected cell differentiates in order to create a replication competent environment, which allows genome amplification and packaging into infectious particles. To do this, they have evolved proteins (E6, E7, and E5) that can interfere with the normal cell cycle regulation and can prevent apoptosis as a result of unscheduled DNA replication. In contrast to low‐risk HPV types, high‐risk Alpha papillomaviruses not only drive cell cycle entry in the upper epithelial layers, but have E6 and E7 proteins that can stimulate the proliferation of infected basal cells and also cause neoplasia. These additional characteristics reflect differences in the viral proteins but also differences in the way that the viral proteins are expressed within the lesion. It is generally accepted that deregulated expression of these cell cycle regulators underlies neoplasia and the eventual progression to cancer in individuals who cannot resolve infection. Most work to date has focused on the study of high‐risk HPV types such as HPV 16 and 18, but in the future, there will be a need to understand the different risks associated with other members of the high‐risk group and to more fully understand the molecular pathways that they subvert. Such approaches will, with some certainty, lead us eventually to the development of better strategies for disease treatment (i.e. targeted antivirals or immunotherapeutics), which are a necessary complement to current methods of disease management (i.e. prophylactic vaccination, screening, surgical ablation, or local immune modulation). In the coming years, it will also be important to consider high‐risk HPV‐associated diseases at sites other than the cervix (e.g. tonsils, other transformation zones) and to understand the mechanisms by which low‐risk HPV types can give rise to papillomatosis and under certain situations even cancers. An important part of these future studies will be to develop our understanding of the Gamma and Beta HPV types at the level of their natural history and to consider the different mechanisms by which this group of viruses cause disease and, in some situations also, cancer.

## Conflict of Interest

The authors have no competing interest.
